# Supplementary material for: Panobinostat Synergistically Enhances the Cytotoxicity of Microtubule Destabilizing Drugs in Ovarian Cancer Cells
Source: Int J Mol Sci. 2022 Oct 27;23(21):13019. doi: 10.3390/ijms232113019 (PMC9657298; doi:10.3390/ijms232113019)
Supplement: Supplementary file 1 [file ijms-23-13019-s001.zip › ijms-1940514-supplementary.pdf]

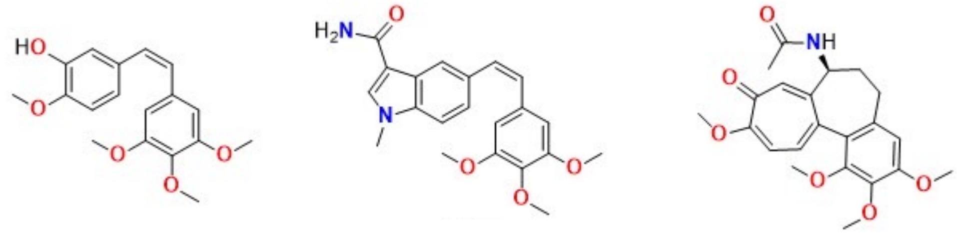

Combretastatin A4 (CA4) **PILA9 (P9)** **Colchicine (COL)**

Figure S1. Chemical structure of combretastatin A4, **PILA9** and colchicine

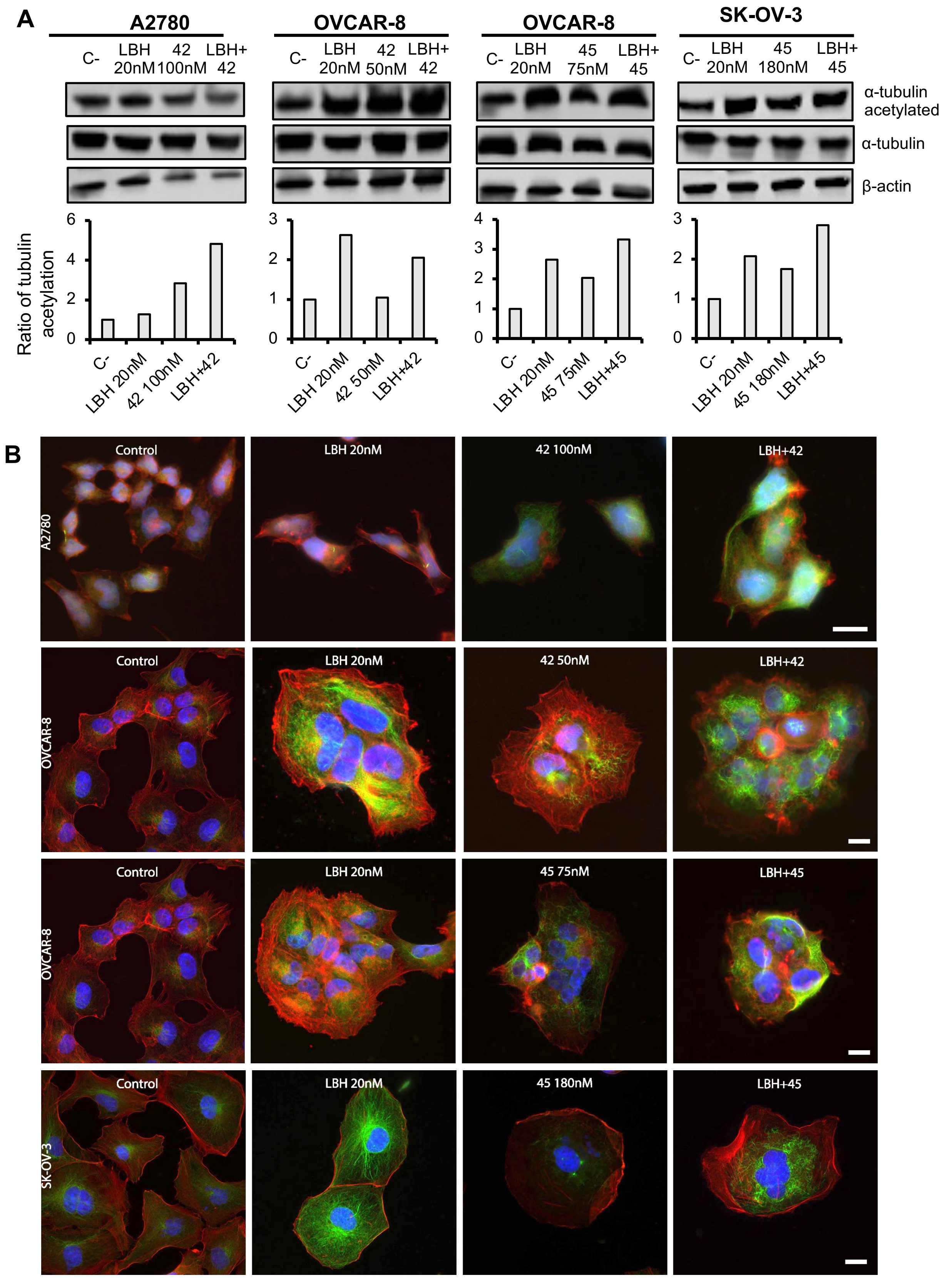

Figure S2. The combination of LBH and **42/45** induces strong acetylation of α-tubulin. A) A2780, OVCAR-8 or SK-OV-3 cells were treated or not with LBH (20nM) and/or the indicated doses of **42/45** for 24 h and the acetylation of α-tubulin were observed by flow western blot. β-actin was used as a loading control. Protein levels were quantified using ImageJ and the ratio of tubulin acetylation was calculated as the proportion of α-tubulin acetylated to α-tubulin total. Previously, these values were normalized using β-actin levels and untreated cells (C-) levels were taken as 100. B) A2780, OVCAR-8 and SK-OV-3 cells were treated or not with LBH and/or **42/45** and α-tubulin acetylated (green) and actin (red) were observed by immunofluorescence. Nuclei were stained with Hoechst (blue). Scale bar: 10 μm.
